# Upregulation of Glucose Uptake and Hexokinase Activity of Primary Human CD4+ T Cells in Response to Infection with HIV-1

**DOI:** 10.3390/v10030114

**Published:** 2018-03-07

**Authors:** Maia Kavanagh Williamson, Naomi Coombes, Florian Juszczak, Marios Athanasopoulos, Mariam B. Khan, Thomas R. Eykyn, Ushani Srenathan, Leonie S. Taams, Julianna Dias Zeidler, Andrea T. Da Poian, Hendrik Huthoff

**Affiliations:** 1 Department of Infectious Diseases, King’s College London, Guy’s Hospital, London SE1 9RT, UK; maia.kavanagh_williamson@kcl.ac.uk (M.K.W.); mathanasop.bio@gmail.com (M.A.); mariamkhan_13@hotmail.co.uk (M.B.K.); 2 Institute of Infection & Global Health, University of Liverpool, 8 W Derby St., Liverpool L7 3EA, UK; naomi.coombes@liv.ac.uk; 3 Faculty of Medicine and Pharmacy, University of Mons, Place du Parc 20, 7000 Mons, Belgium; florian.juszczak@umons.ac.be; 4 Division of Imaging Sciences and Biomedical Engineering, The Rayne Institute, St. Thomas’ Hospital, King’s College London, Westminster Bridge Road, London SE1 7EH, UK; thomas.eykyn@kcl.ac.uk; 5 Centre for Inflammation Biology and Cancer Immunology (CIBCI), School of Immunology & Microbial Sciences, Department of Inflammation Biology, King’s College London, London SE1 1UL, UK; ushani.srenathan@kcl.ac.uk (U.S.); leonie.taams@kcl.ac.uk (L.S.T.); 6 Instituto de Bioquímica Médica Leopoldo de Meis, Universidade Federal do Rio de Janeiro, Rio de Janeiro 21941-902, Brazil; juliannaze@gmail.com (J.D.Z.); dapoian@bioqmed.ufrj.br (A.T.D.P.)

**Keywords:** HIV-1, glycolysis, GLUT1, GLUT3, GLUT4, GLUT6, hexokinase, HK1, T lymphocytes

## Abstract

Infection of primary CD4+ T cells with HIV-1 coincides with an increase in glycolysis. We investigated the expression of glucose transporters (GLUT) and glycolytic enzymes in human CD4+ T cells in response to infection with HIV-1. We demonstrate the co-expression of GLUT1, GLUT3, GLUT4, and GLUT6 in human CD4+ T cells after activation, and their concerted overexpression in HIV-1 infected cells. The investigation of glycolytic enzymes demonstrated activation-dependent expression of hexokinases HK1 and HK2 in human CD4+ T cells, and a highly significant increase in cellular hexokinase enzyme activity in response to infection with HIV-1. HIV-1 infected CD4+ T cells showed a marked increase in expression of HK1, as well as the functionally related voltage-dependent anion channel (VDAC) protein, but not HK2. The elevation of GLUT, HK1, and VDAC expression in HIV-1 infected cells mirrored replication kinetics and was dependent on virus replication, as evidenced by the use of reverse transcription inhibitors. Finally, we demonstrated that the upregulation of HK1 in HIV-1 infected CD4+ T cells is independent of the viral accessory proteins Vpu, Vif, Nef, and Vpr. Though these data are consistent with HIV-1 dependency on CD4+ T cell glucose metabolism, a cellular response mechanism to infection cannot be ruled out.

## 1. Introduction

The CD4+ T cell compartment is an essential component of the adaptive immune system that regulates the initiation or suppression of humoral, as well as cellular immune responses, and the establishment of immunological memory. CD4+ T cells are also the primary target for infection with the human immunodeficiency virus type 1 (HIV-1), and their depletion heralds the onset of acquired immunodeficiency syndrome (AIDS) [1]. HIV-1 is known to productively infect activated CD4+ T cells, but resting cells are refractory to infection [2,3,4,5,6]. The transition from quiescent to activated effector CD4+ T cells requires increased biosynthesis and energy to support cell growth, proliferation, and expression of effector molecules, which is achieved through a metabolic switch upon T cell receptor (TCR) stimulation and co-stimulation. In particular, CD4+ T cell activation coincides with increased glucose and amino acid uptake, as well as the upregulation of both glycolysis and glutaminolysis [7,8]. TCR stimulation and co-stimulation of CD28 leads to signaling through AKT (Protein Kinase B), PI3K (Phosphatidylinositol 3-Kinase), mTOR (mammalian Target of Rapamycin), and ERK (Extracellular signal Regulated Kinase), that results in increased expression and targeting of nutrient transporters to the cell membrane [9,10,11,12]. Furthermore, a Myc-driven transcriptional program ensures that metabolic enzymes and nutrient transporters are coordinately expressed upon CD4+ T cell activation [13]. Glucose metabolism is particularly important to support the biosynthesis of effector cytokines, as in the absence of flux through the glycolytic pathway, the enzyme GAPDH acts as a translational repressor of mRNAs, including interferon gamma (IFNγ) and IL-2 [14].

We previously reported an important role for glucose metabolism in supporting HIV-1 replication and pathogenesis that was manifested by increased glycolytic activity of HIV-1 infected cells and reduced viral output, as well as apoptotic cell death upon substitution of glucose with galactose as a carbohydrate nutrient [15]. Import of glucose into activated T cells is known to involve the hexose transporter GLUT1, and its expression and translocation to the cell membrane is well documented to occur in an activation-dependent manner [16,17,18]. Likewise, an important role has been attributed to GLUT1 in supporting HIV-1 replication [19] and as a marker of HIV-1 induced chronic inflammation [20,21,22].

GLUT1 is a ubiquitously expressed uniporter that facilitates transport of glucose and other hexoses across the plasma membrane. In humans, the protein family of glucose transporters consists of 14 members, known as GLUT or SLC2A (solute carrier 2A) 1 to 14, many of which have as yet undefined tissue and substrate specificity, transport kinetics, and subcellular localization [23,24]. GLUT1 has been extensively investigated in relation to T cell activation and it has been suggested that the relative abundance of GLUT1 on the cell surface defines thymocyte differentiation [25], the identity of CD4+ and CD8+ effector subsets [26], memory cells [27] and regulatory T cells (Tregs) [28,29]. GLUT1 overexpression in a transgenic mouse model increased the expression of cytokines by effector T cells (Teff) and the incidence of inflammatory events [27,30]. There have been sporadic studies reporting the detection of GLUT3 [31,32,33] or GLUT3 and GLUT4 [34] in whole lymphocyte preparations, and more recently that GLUT3 is expressed in the murine Th17 effector subset of CD4+ T cells [29]. A recent study investigating the expression of all GLUT mRNAs in murine CD4+ T cells reported activation-induced increases in GLUT1 and GLUT6 mRNA, whereas GLUT3 and GLUT8 mRNA were downregulated [35]. Thus, there is compelling evidence to indicate that activated T cells do not rely exclusively on GLUT1 to support the metabolic switch associated with activation. The full repertoire of GLUT proteins expressed by human CD4+ T cells upon activation and infection with HIV-1 has, to date, remained unexplored. We here report that human CD4+ T cells show activation-dependent upregulation of GLUT1, GLUT3, GLUT4, and GLUT6 mRNA as well as protein, and that GLUT protein expression is further enhanced upon infection of the cells with HIV-1. Using inhibitors with differential specificity for GLUT proteins, we provide evidence that glucose transporters other than GLUT1 contribute to supporting CD4+ T cell glucose uptake during activation.

After the uptake of glucose into the cell, the hexokinase enzymes commit it to metabolic processing by phosphorylation to glucose-6-phosphate, which can either enter glycolysis or the pentose phosphate pathway. Upon activation, hexokinase activity in T cells is highly upregulated [36]. In humans, four distinct isozymes of hexokinase have been described [37], but their roles in human T cell activation remain largely unstudied. In mice, the activation-responsive expression of hexokinase isozymes I, II, and III has been reported, with HK1 being downregulated, and HK2 and HK3 upregulated upon activation [38]. In humans, the expression of HK1 mRNA [39] and HK2 protein [40] in activated T cells has been reported. Whilst there is mounting evidence from studies of metabolite abundances and lactate secretion that glycolysis is upregulated in HIV-1 infected T cells [15,41,42], the modulation of enzyme activities in the pathway upon infection with HIV-1 has not previously been investigated. By assessing the activity of all glycolytic enzymes in uninfected and HIV-1 infected human CD4+ T cells, we identified a marked increase in hexokinase activity in the HIV-1 infected cells. We show that in human CD4+ T cells, both HK1 and HK2 are upregulated in an activation-dependent manner, whilst HK3 and HK4 are not expressed in resting or activated cells. Furthermore, the expression of HK1 is further increased in HIV-1 infected CD4+ T cells, whereas HK2 is not.

## 2. Materials and Methods

### 2.1. Cells and Culture

The primary human CD4+ T cells used in this study were isolated from leucocyte cones obtained from the UK National Health Service (NHS) National Blood Service. Peripheral blood mononuclear cells (PBMCs) were isolated by density centrifugation using Lymphoprep (StemCell Technologies, Vancouver, BC, Canada) and CD4+ T cells (including CD45RA+ and CD45RO+) were isolated by magnetic activated cell sorting (MACS) with the appropriate kits from Miltenyi Biotec, according to the manufacturer’s instructions. The purity of the resulting CD4+, CD45RA+, and CD45RO+ T cell populations was determined by flow cytometry, and was consistently ≥95%. Cells were immediately used for downstream analyses. T cells were activated with CD3/CD28 T-cell activator Dynabeads (12.5 μL per 10^6^ cells) and cultured in Roswel Park Memorial Institute Media (RPMI-1640) containing 10% FBS, 1% penicillin/streptomycin, and 15 international units/mL IL-2 (all Invitrogen, Carlsbad, CA, USA). Experiments addressing signaling through the PI3K/mTOR pathways were conducted in the presence of 10 mM LY294002, 20 nM rapamycin, or 1 mM PP242 (all from Sigma, St. Louis, MO, USA). CD4+ T cells that were cultured under Th17-polarising conditions were cultured with plate-bound α-CD3 (1.25 μg/mL) (Biolegend, Cambridge, UK), soluble α-CD28 (1 μg/mL) (BD Biosciences, Franklin Lakes, NJ, USA), hrIL-1β (10 ng/mL), and hrIL-23 (20 ng/mL) (Peprotech, Rocky Hill, NJ, USA) for 3 days. After culture, CD4+ T cells were stimulated with PMA (50 ng/mL) and ionomycin (750 ng/mL) (Sigma) for 1.5 h, and labelled using the IL-17 Cytokine Secretion kit (Miltenyi Biotec, Bergisch Gladbach, Germany), according to the manufacturer’s instructions. For experiments addressing the inhibition of transcription, actinomycin D (Sigma, St. Louis, MO, USA) was added to the cultures at a final concentration of 1 μg/mL immediately prior to activation.

### 2.2. Virus Production and Infections

Primary CD4+ T cells were either infected with wild type (wt) NL4.3 HIV-1 or with non-infectious Env-deleted NL4.3 (∆Env) preparations that were produced by PEI transfection of HEK293T cells. All virus stocks were generated by filtering of HEK293T cell supernatant through a 0.45 mm cellulose membrane (Millipore, Darmstadt, Germany) and subsequent ultracentrifugation over a 20% sucrose cushion. Virus pellets were resuspended in glucose-free Dulbecco’s Modified Eagle’s Medium (DMEM), and virus infectivity was then determined by titration on CEM-ss cells, with subsequent intracellular p24^Gag^ staining and flow cytometry analysis. The volume of viral stock needed to achieve the desired percentage of infected cells was then calculated for each experiment. For infection experiments, primary CD4+ T cells were activated with anti-CD3/CD28 Dynabeads in RPMI with IL-2 for four days prior to infection. Jurkat and CEM-ss cells were infected in RPMI only. Some cultures were pretreated with nevirapine (10 µM, Sigma) for 1 h prior to infection, or infected for 24 h, at which point nevirapine was added to limit infections to a single round.

### 2.3. Extracellular Flux Measurements

Oxygen consumption and extracellular acidification rates, OCR and ECAR respectively, were measured on an XF24 apparatus from Seahorse Biosciences, as described previously (25). Briefly, all experiments were carried out in filter-sterilized DMEM reconstituted from powder formulation (Sigma, St. Louis, MO, USA) supplemented with 1 mM glutamine, but without sodium bicarbonate, phenol red, or serum, at a pH between 7.2 and 7.4 at 37 °C. Cells were seeded at a density of 1.5 to 2.0 × 10^5^ per well by centrifugation in XF24 culture plates that were coated with poly-d-Lysine (Sigma). Glucose, 2-deoxyglucose (Sigma) and glucose transporter inhibitors (STF-31, GTI-2, and WZB117 from Merck; ritonavir and indinavir from Sigma) were injected to the indicated final concentrations. In each experiment, 6 wells were dedicated to each inhibitor concentration, 4 for dimethyl sulfoxide (DMSO) controls and 2 for background detection; titrations over six different concentrations were therefore conducted on two separate XF24 plates and cartridges for each donor. Data are represented as the average with error bars indicating the standard deviation.

### 2.4. ELISA and Western Blotting

Cytokine concentrations in the supernatant from pre-sorted CD4+ T cells, cultured at a density of 10^6^ cells/mL, were determined by the use of the IL-4, IFNγ, and IL-17A ELISAMAX kits from Biolegend, according to the manufacturer’s instructions. Determination of the amounts of p24^Gag^ in infected cultures or virus stocks was performed with the p24^Gag^ ELISA kit from Perkin Elmer according to the manufacturer’s instructions. For Western blotting, samples of typically 2 × 10^6^ cells were resuspended in 50 μL of TBS buffer with 2% SDS, complete protease inhibitor cocktail (Roche, Basel, Switzerland) and 0.5 mM PMSF. Samples were incubated with Benzonase (Sigma) for 20 min at room temperature, the lysate was added to 10 μL of 6× Laemmli buffer, heated for 10 min at 50 °C, resolved by PAGE on 11% gels, transferred onto nitrocellulose membranes, and detected with fluorescent secondary antibodies using the Licor Odyssey imaging system. The following antibodies were used according to the manufacturer’s instructions: GLUT1 (Abcam Ab40084, Cambridge, UK), GLUT3 (Santa Cruz G-5 and Abcam Ab191071), GLUT4 (Santa Cruz IF8, Dallas, TX, USA), GLUT6 (Santa Cruz B-3), GLUT8 (Santa Cruz H-60 and M-18), GLUT9 (Santa Cruz C-16 and Thermo Scientific PA5-22971), GLUT10 (Santa Cruz H-96), GLUT11 (Santa Cruz G-20), GLUT12 (Santa Cruz P-13), GLUT13 (Fitzgerald 70R-GR005, Acton, MS, USA), HK1 (GeneTex GTX82790, Hsinchu City, Taiwan), HK2 (Abcam Ab104836), HK3 (GeneTex GTX107882), HK4 (GeneTex GTX111517), VDAC1 (Abcam Ab14734), ENO1 (GeneTex GTX113179), G6PDH (Abcam Ab133525), GPI (GeneTex GTX113203), GFPT1 (Abcam AB125069), GAPDH (Sigma 71.1, Abcam Ab8245 and Ab9845), Tubulin (Abcam Ab7291) and HSP90 (Santa Cruz H-114 and 4F10). The specificities of GLUT antibodies were confirmed by the following observations: the unique detection of GLUT6 expressed from the OriGene plasmids RC204391 and RC227402 with the B-3 antibody after transfection of 293T cells, and the unique detection of GLUT3 in 293T cells [43] with the G-5 and Ab191071 antibodies [44]. The specificity of the IF8 antibody towards GLUT4 has been reported previously [45,46]. HIV-1 Gag and Vif were detected using supernatants from the mouse hybridoma cell lines 183-H12-5C and 319, respectively, and were kind gifts from M. Malim. HIV-1 Vpr (11836), Vpu (112942), and Nef (1539) antibodies were obtained from the NIH AIDS reagent program.

### 2.5. Flow Cytometry

Cells were washed with FACS Buffer (PBS with 1% PenStrep and 2% FBS) and, if necessary, permeabilized and fixed with the Cytofix/Cytoperm reagents from BD biosciences according to the manufacturer’s instruction. Cells were then incubated with antibodies according to manufacturer’s instructions, washed twice with FACS Buffer, and resuspended in FACS Buffer before being acquired on a FACSCantoII (BD) for analytical flow cytometry, and on a FACSAriaII (BD) for cell sorting. For the determination of cell death, CD4+ T cells were harvested and resuspended in PBS with LiveDead stain (Life technologies, Carlsbad, CA, USA) 1:1000 for 30 min in the dark at room temperature. All flow cytometry data were analyzed on FlowJo software (FLowJo, Ashland, OR, USA). The following antibodies were used: GLUT1-RBD-GFP (Metafora), CD69 (APC: BD 555533), CD45RA (FITC: Biolegend H1100), CD45RO (PB: Biolegend UCHL1), CD4 (PercP/Cy5.5: Biolegend SK3), CCR6 (BV510 Biolegend GO34E3), CD127 (BV605: Biolegend A01905), CD25 (PE: Biolegend M-A251), CCR4 (PE/Cy7: Biolegend L291H4), CD3 (PE/Cy7; Biolegend UCHT1), and CD14 (APC/Vio770: Miltenyi TUK4). HIV-1 infected samples were assessed for p24^Gag^ and CD4 expression using the KC57-RD1 (Beckman Coultier, Brea, CA, USA) and RPA-T4 (BD biosciences) antibodies, respectively. For intracellular staining of p24^Gag^, cells were treated with trypsin to remove surface bound HIV-1, and permeabilized with the Cytofix/Cytoperm kit (BD Biosciences), according to the manufacturer’s instructions. Staining of infected cells with CD4 antibody followed fixing of the cells with 4% PFA.

### 2.6. qPCR

RNA was extracted from cells using the QIAGEN (Hilden, Germany) miRNeasy Mini kit according to the manufacturer’s instructions, including the RNase-free DNase treatment to eliminate genomic DNA contamination. The NanoDrop ND-100 Spectrophotometer was used to measure the optical density at 230, 260, and 280 nm to quantify and assess RNA quality. Equal amounts of RNA from samples for each donor were converted into cDNA using the High-Capacity cDNA Reverse Transcription Kit from Applied Biosystems with the following conditions: 25 °C/10 min; 3 °C/120 min; 4 °C/∞. Quantitative PCR (qPCR) was performed with the cDNA using TaqMan universal PCR master mix and specific primer/probe sets from Life Technologies. All primer pairs were selected to cross exon–exon junctions as an additional measure to prevent contamination from genomic DNA. Reactions were carried out in a final volume of 10 μL, and performed in triplicate with the following conditions: 95 °C/10 min; (95 °C/15 s, 60 °C/1 min) × 40 cycles. Control reactions in the absence of cDNA consistently yielded no signal during the amplification cycles. The comparative ΔΔCt method was used to calculate changes in the mRNA abundance normalized to the control genes 18S, B2M, and EIF4A2 for activated compared with resting cells. The final fold change was calculated as the geometric mean of the values obtained with the three control genes.

### 2.7. ^1^H-NMR Analysis

Culture supernatants were filtered through 0.45 μm nitrocellulose, and methanol added to a final concentration of 75%. Debris was removed by centrifugation on an Eppendorf benchtop centrifuge at maximum speed for 10 min at 4 °C. The liquid phase was transferred to a clean Eppendorf tube, dried in a speedvac at 30 °C, and resuspended in 600 µL phosphate-buffered deuterium oxide (D_2_O containing 8 g/L NaCl, 0.2 g/L KCl, 1.15 g/L Na_2_HPO_4_, 0.2 g/L KH_2_PO_4_, and 0.0075% w/v trimethylsilyl propanoic acid as an internal reference). Samples were acquired by ^1^H-NMR using a Bruker Advance III 400 MHz (9.4 T) wide-bore spectrometer (Bruker, Billerica, MA, USA) with a high-resolution broadband spectroscopy probe at 298 K. Fully relaxed ^1^H-NMR spectra were acquired using a NOESY (nuclear Overhauser effect spectroscopy) 1D pulse sequence with 128 scans, 2 dummy scans, spectral width of 14 ppm, 32,768 data points, a repetition time of 5 s per scan, and a total experiment duration of 10 min. Spectra were baseline-corrected, and peak integrals quantified with respect to the 1% ^13^C TSP peaks as the internal reference, and analyzed using TopSpin software version 2.1 (Bruker). Metabolite identity was determined from previously acquired data as well as publicly available reference spectra. Peak integrals from HIV-1 infected or ∆Env uninfected samples were subtracted from that of their paired “media only” sample to determine uptake and secretion of metabolites from or to the media, and normalized to cell number at the end of the experiment. Significance of the difference in metabolite abundance was assessed by *t*-test (*p* < 0.05).

### 2.8. Glycolytic Enzyme Kinetic Assay

Cells (20 × 10^6^) were washed three times in PBS, and lysed in a buffer consisting of 10 mM Tris, 0.25 mM sucrose, 20 mM sodium fluoride, 5 mM EDTA, 0.5% Triton-X, 10% glycerol, cOmplete protease inhibitors (Roche) at pH = 7.4, and stored at −80 °C until the day of analysis. The final reaction conditions for the assessment of glycolytic enzymes were as follows: HK (50 mM Tris-HCL pH = 7.4, 5 mM MgCl_2_, 2 mM sodium azide, G6PDH 1 U/mL, 0.5 mM β-NADP+, 0.1% Triton, 1 mM ATP, 10 mM glucose), GPI (50 mM Tris-HCL pH = 7.4, G6PDH 1U/mL, 0.5 mM β-NADP+, 1 mM fructose-6-phospate), PFK (100 mM Tris-HCL pH = 7.4, 5 mM MgCl_2_, 5 mM NH_4_SO_4_, aldolase 1.5 U/mL, TPI 3.2 U/mL, GPDH 1 U/mL, 0.1 mM ATP, 0.25 mM β-NADH, 1 mM fructose-6-phospate), aldolase (50 mM Tris-HCL pH = 7.4, TPI 3.2 U/mL, GPDH 3.2 U/mL, 5 mM fructose-1,6-biphosphate, 0.3 mM β-NADH), TPI (100 mM Tris-HCL pH = 7.4, GPDH 2 U/mL, 0.4 mM β-NADH, 0.6 mM glyceraldehyde-3-phosphate), GAPDH (50 mM Tris-HCL pH = 7.4, 2 mM MgCl_2_, 1 mM ATP, 1 mM EDTA, PGK 13 U/mL, 0.25 mM β-NADH, 5 mM 3-phosphoglycerate), PGK (50 mM Tris-HCL pH = 7.4, 2 mM MgCl_2_, 1 mM ATP, 1 mM EDTA, GAPDH 5 U/mL, 0.25 mM β-NADH, 5 mM 3-phosphoglycerate), PGM (100 mM Tris-HCL pH = 7.4, 5 mM MgCl_2_, 3 mM ADP, 1 mM EDTA, PK 4 U/mL, LDH 8 U/mL, ENO 1.4 U/ML, 0.3 mM β-NADH, 5 mM 3-phosphoglycerate), ENO (100 mM Tris-HCL pH = 7.4, 10 mM MgCl_2_, 2 mM ADP, PK 5 U/mL, LDH 5 U/mL, 0.4 mM β-NADH, 1 mM 2-phosphoglycerate), and PK (Imidazole 50 mM, 100 mM KCl, 2 mM MgCl_2_, 1 mM ADP, LDH 55 U/mL, 0.25 mM β-NADH, 1 mM phosphoenolpyruvate). All fine chemicals and enzymes for these assays were purchased from Sigma. Spectrophotometric analysis of enzyme kinetics was measured as changes in absorption at λ340 nm. For each analysis, the reaction components were combined into 1 mL cuvettes and placed in the spectrophotometer. Absorption was measured for at least 10 s to ensure the reading was stable. Cell lysates were added by applying to a small plastic spatula, and immersing in the cuvette. Absorption was measured for at least 30 s. Following acquisition of the data, the slope of the absorbance over time was calculated, and the activity of the enzyme calculated using the following equation:

enzyme specific activity (μg substrate/10^6^/min) = (ΔAbs/min × reaction volume (μL))/(NADH molar extinction coefficient × cell number (10^6^)) (1)

BCA assays (Pierce) were conducted on each lysate sample, and the enzyme activity was then normalized to protein content of the samples.

### 2.9. Statistical Analyses

Average values ± the standard deviation are shown throughout, except on logarithmic scales where only the + standard deviation is indicated. Significance was calculated using the two-tailed paired *t*-test with GraphPad Prism 7 software (San Diego, CA, USA).

### 2.10. Ethical Approval

Cultured cell studies using blood obtained from healthy human volunteers (UK National Blood Service) was approved on the 3rd of February 2006 by the Guy’s Hospital Research Ethics Committee (Ref: 03/02/06). Subjects were controlled in accordance with the tenets of the Declaration of Helsinki.

## 3. Results

### 3.1. Activation-Dependent Expression of Multiple GLUT Proteins in Human CD4+ T Cells

**Figure 1:**
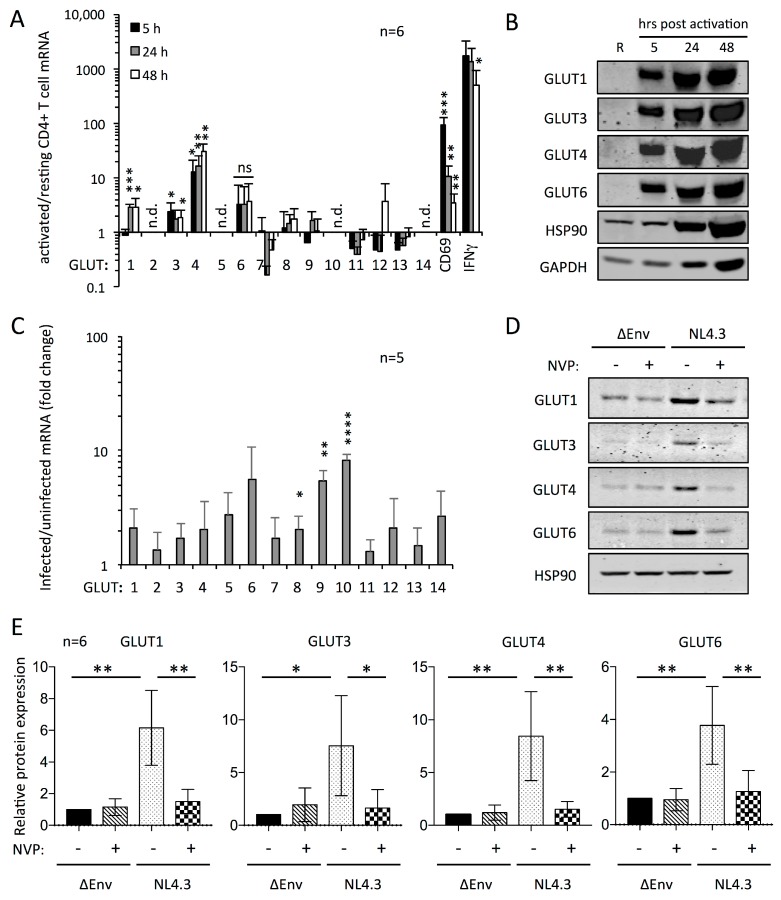
Multiple GLUT proteins are expressed in activated human CD4+ T cells and upregulated in response to infection with HIV-1. (**a**) The abundance of GLUT1-14, CD69, and IFNγ mRNA in activated relative to resting CD4+ T cells at 5, 24, and 48 h after activation from six donors as determined by qPCR: n.d. indicates not detected. The data are averages after normalization to three reference genes (18S, B2M, and EIF4A2), with error bars representing the standard deviation; (**b**) Representative Western blotting analysis showing the detection of GLUT proteins in equal numbers of resting (R) and activated human CD4+ T cells at 5, 24, and 48 h post stimulation with GLUT-specific antibodies. Blots for GAPDH and HSP90 are included as controls; (**c**) The relative abundance of GLUT1-14 mRNA in HIV-1 infected versus uninfected cells as determined by qPCR of samples from 5 donors; (**d**) Representative Western blotting analysis of the expression of GLUT1, 3, 4, and 6 of uninfected (ΔEnv) and HIV-1 NL4.3 infected primary CD4+ T cells in the presence or absence of nevirapine (NVP). Equal numbers of infected and uninfected cells were used, and HSP90 is shown as the loading control; (**e**) Quantified data from Western blotting analysis shown in (**d**) from six different donors. Significance is indicated by * *p* ≤ 0.05, ** *p* ≤ 0.01, *** *p* ≤ 0.001, and **** *p* ≤ 0.0001. For qPCR data, significance is indicated for upregulated transcripts only, n.d. incicates not detected and ns indicated not statistically significant.

We previously reported moderate upregulation of intracellular and surface expressed GLUT1 in response to infection of primary human CD4+ T cells with HIV-1, as assessed by flow cytometry [15]. Because those experiments made use of HIV-1 molecular clones expressing fluorescent proteins, we could not rule out that the increase in GLUT1 expression observed by flow cytometry was influenced by some overlap of the GLUT1 and GFP/dsRED signals. In addition, a recent study reported the expression of other glucose transporters in addition to GLUT1 in murine CD4+ T cells [35]. We therefore sought to assess the full range of glucose transporters expressed by human CD4+ T cells and their modulation upon infection with HIV-1. To assess the expression of all GLUT family members in resting versus activated human CD4+ T cells, we first performed a qPCR analysis with GLUT-specific primers using the ΔΔCt method, with CD69 and IFNγ as controls for T cell activation, and the geometric mean of the housekeeping genes B2M, 18S, and EIF4A2 serving as the normalization standard (Figure 1A). We assessed mRNA extracted from purified CD4+ T cells from five different donors at 5, 24, and 48 h post stimulation with anti-CD3/CD28-antibody-coated beads in the presence of IL-2, in addition to a sample of resting cells. We consistently observed upregulation of GLUT1, GLUT3, GLUT4, and GLUT6 mRNA in activated, compared with resting cells. This was highly significant for GLUT1, GLUT3, and GLUT4, but failed to reach statistical significance for GLUT6. We note that we observed upregulation of GLUT6 mRNA in each case, but donor variability prevented results from being considered significant. GLUT1 mRNA upregulation was not apparent at 5 h after stimulation, but reached an average three-fold induction compared with resting cells at 24 and 48 h after activation. In addition, we observed an average two- to three-fold increase in expression of GLUT3 and GLUT6 that was already apparent at 5 h after activation. The most pronounced upregulation was observed with GLUT4 mRNA, which was twelve- to thirty-fold increased in a time-dependent manner from 5 to 48 h after activation, respectively. While some mRNA for GLUT7, 8, 9, 11, 12, and 13 was detectable, none of these reached two-fold upregulation that was consistent across the different donors. The mRNAs of GLUT2, 5, 10, and 14 were undetectable throughout our analyses. As a complementary approach, we subjected cell lysates from the same samples to Western blotting with antibodies specific for individual GLUT proteins (Figure 1B and Supplementary Figure S1A; see also Materials and Methods for antibody specificity). In agreement with the qPCR analysis, we detected activation-induced upregulation of GLUT1, GLUT3, GLUT4, and GLUT6 that was apparent at 5 h post activation, and remained so at 24 h and 48 h post activation. Importantly, those GLUT proteins that did not show any upregulation of the mRNA were also not detected by Western blotting (Figure S1A). We note that the increase in the levels of GAPDH and HSP90 protein observed in the Western blots at various time points after activation reflects the widespread increase in gene expression after activation of CD4+ T cells [47,48], as equal numbers of cells were present in each sample. However, the upregulation of GLUT1, 3, 4, and 6 proteins preceded and exceeded that of GAPDH and HSP90 within the first 5 h after activation (Figure 1B and Figure S1B), indicating that this is a specific early activation-induced process, as opposed to more general stimulation of gene expression. At 5 h after activation, the increase in GAPDH and HSP90 protein expression remained below 1.5-fold, while the average enhancement of GLUT proteins was between 20- and 100-fold.

We further investigated the response of GLUT protein expression to activation in time course experiments that included sampling as early as 15 min after activation (Figure S1B). The Western blotting analysis revealed that the expression of GLUT1, 3, 4, and 6 was detectable at abundant levels within 15 min of activation. This rapid expression of the GLUT proteins, in addition to the observation of only moderate upregulation of some of the GLUT mRNAs early after activation (Figure 1A), may indicate some degree of translational regulation. We therefore assessed the effect of the transcription inhibitor actinomycin D on protein, as well as mRNA levels, at 24 h after activation (Figure S1C). The activation-dependent expression of GLUT1, 3, 4, and 6 proteins was unaffected by the presence of actinomycin D, but it did result in the ablation of HSP90 expression, as well as the reduction of GAPDH expression to levels similar to those observed in resting CD4+ T cells (Figure S1C). Analysis of the corresponding mRNA levels confirmed that transcription of the GLUT genes, as well as IFNγ and GAPDH, were inhibited upon provision of actinomycin D, though this only reached significance for GLUT1 mRNA (Figure S1D). We note that this experiment was normalized to the 18S reference gene only, as the other reference genes used throughout our work (B2M and EIF4A2) were also strongly inhibited by actinomycin D. Although the 18S RNA levels were more robust in the presence of actinomycin D, moderate inhibition of 18S transcription was also apparent. This contributed to the relatively minor differences between actinomycin D treated and untreated cells for GLUT3 and GLUT4, whose mRNA levels were quite constant in either condition. Together, these data indicate that translational regulation is an important component alongside transcriptional regulation in the activation-dependent expression of glucose transporters in human CD4+ T cells. In agreement with previous work on murine CD4+ T cells [35], targeting of GLUT1 to the membrane of CD4+ T cells was only partially inhibited by pharmacological blocking of the PI3K/mTOR pathway using the drugs Rapamycin (mTOR), PP242 (mTOR), and LY294002 (PI3K), while intracellular expression levels of GLUT1, 3, 4, and 6 were unaffected (Figure S1E,F).

We also compared the expression of GLUT proteins in naïve versus memory CD4+ T cells after isolation of CD45RA+ and CD45RO+ cell populations, respectively (Figure S2). Samples were harvested at 5 h, 24 h, and 48 h after activation, and subjected to analysis by qPCR, Western blotting, and flow cytometry for cell surface expression of GLUT1 as well as the activation marker CD69 (Figure S2A–D). The identity of GLUT proteins expressed in the CD45RA+ and CD45RO+ populations was the same, being GLUT1, GLUT3, GLUT4, and GLUT6, with the other GLUT members not being detected or upregulated, in accordance with the data obtained with CD4+ T cells (Figure 1A). The qPCR analysis yielded no significant differences in the relative upregulation of GLUT mRNA between CD45RA+ and CD45RO+ cells (Figure S2A). Western blotting also revealed comparable levels of the GLUT proteins in the CD45RA+ and CD45RO+ populations at 5, 24, and 48 h (Figure S2B). Flow cytometry analyses demonstrated that the cell surface expression of CD69 was greater for CD45RO+ cells at 5 h after activation compared with the CD45RA+ cells, which would be expected for cells of the memory versus naïve phenotype (Figure S2C–E). Likewise, flow cytometry analysis of surface-expressed GLUT1 revealed that its cell surface occupancy was significantly greater on CD45RO+ cells than on CD45RA+ cells at 24 and 48 h after activation (Figure S2D,E). Our data therefore suggest that expression of GLUT1 on the cell surface is more rapid on CD4+ T cells of the memory phenotype compared with naïve cells. We note that GLUT1 is the only glucose transporter for which there exists a cell surface-specific flow cytometry reagent, which prevented the analysis of surface expression of GLUT3, 4, and 6.

We next investigated the expression of the various GLUT proteins in human Th1, Th2, and Th17 effector cells compared with CD45RA+ cells. Cells were sorted based on expression of chemokine receptors in a manner that predisposes them to differentiation towards Th1, Th2, and Th17 phenotypes [49], and those effector phenotypes were confirmed by assessment of cytokines present in the culture supernatant using ELISA assays for IFNγ, IL-4, and IL-17 (Figure S3A,B). The sorted Th effector cells were activated and cultured for 48 h, and analyzed for the expression of GLUT mRNA by qPCR (Figure S3C). There was no significant difference in the expression of different GLUT family members between the Th subsets as determined by the upregulation of mRNA at 48 h after activation, with the exception of significantly lower GLUT4 mRNA in Th1 cells. A slightly higher activation-induced upregulation of GLUT3 mRNA in the Th17 subset compared with the other subsets was not statistically significant. Because we previously observed that transcriptional upregulation of the GLUT genes in response to activation was generally moderate compared with protein levels in CD4+ T cells, we determined GLUT3 protein expression in CD4+ T cells that were sorted into populations that did or did not express IL-17. Western blotting revealed that those cells expressing IL-17 were moderately enriched for GLUT3 compared with cells not expressing IL-17 (Figure S3D,E). This is in agreement with the reported enrichment of GLUT3 protein in murine Th17 cells [29].

### 3.2. Upregulation of GLUT Proteins in HIV-1 Infected CD4+ T Cells

Having identified the expression of GLUT1, 3, 4, and 6 in activated human CD4+ T cells, we next turned our attention to their expression after the infection with HIV-1. Because we routinely expand the activated CD4+ T cells for a period of up to four days prior to the infection, to obtain enough HIV-1 infected cells for downstream analyses, we first monitored the expression levels of the GLUT proteins over this period of time (Figure S4A). This revealed that the initial increase of GLUT1, 3, 4, and 6 expression levels up to 48 h after activation that we previously observed (Figure 1A,B) was followed by a gradual decline of GLUT expression levels up to day 6. This was not a general feature of protein expression in the activated CD4+ T cells, as activation-dependent expression of HK1, HK2, GAPDH, and HSP90 remained high throughout the six-day period. Typically, we activated human primary CD4+ T cells and expanded them for four days, after which they were infected with HIV-1 NL4.3 or an Env-deleted non-infectious NL4.3 preparation (ΔEnv) for a further 48 h. Samples corresponding to equal numbers of infected and uninfected cells were harvested and subjected to subsequent analysis of the expression of GLUT proteins by qPCR and Western blotting (Figure 1C–E and Figure S4B,C).

The qPCR analysis revealed a moderate upregulation of all GLUT mRNAs in infected cells compared with uninfected ΔEnv-treated control cells (Figure 1C), including those that were undetectable in our previous analysis of resting versus activated cells, such as GLUT2 and GLUT14 (Figure 1A). The detection of those GLUT mRNAs appeared to be due to a general increase in transcriptional activity of the activated CD4+ T cells during the prolonged proliferation for 4 to 6 days prior to the analysis, as indicated by increases in the Ct values of GLUT as well as control genes [44]. We observed a general elevation of GLUT mRNA in HIV-1 NL4.3 infected over uninfected ΔEnv-treated control cells of approximately two-fold, though this was largely within the error of the experiments, and not statistically significant (Figure 1C). Most notably, GLUT6, 9, and 10 mRNA showed a four- to eight-fold enhancement in HIV-1 infected cells over uninfected cells. Of those, we note that the mRNA for both GLUT9 and 10 was present in low abundance, as evidenced by high Ct values [44]; thus, relatively minor increases in their Ct values in infected cells caused a significant fold-change compared with uninfected cells, and a significant upregulation.

To determine if HIV-1 infection caused alterations in the expression of the glucose transporters at the protein level, uninfected and HIV-1 infected cell samples were analyzed by Western blotting (Figure 1D,E and Figure S4B,C). We included samples that were treated with the antiretroviral reverse transcriptase inhibitor nevirapine (NVP) prior to addition of the viral inoculum to assess if any changes in GLUT expression were associated with viral entry or productive infection. We assessed the proportion of infected and uninfected cells in each sample, as well as the effectiveness of inhibiting HIV-1 replication with NVP, by flow cytometric analysis of p24^Gag^ expression and downregulation of CD4 (Figure S4D). Throughout these studies, samples were only included if a minimum of 60% of the cells in the samples treated with wt HIV-1 NL4.3 expressed p24^Gag^. In line with our observation that GLUT9 and 10 mRNA abundances were low in these samples, no GLUT 9 and 10 proteins could be detected by Western blotting (Figure S4B). By contrast, GLUT1, 3, 4, and 6 all demonstrated increased protein expression in infected cells compared with uninfected cells and NVP-treated controls (Figure 1D,E and Figure S4B,C). Upon quantification of protein, these increases reached between four- and seven-fold, and were all highly significant. Thus, our results demonstrate that the expression of GLUT1, 3, 4, and 6 proteins is upregulated in HIV-1 infected cells in a manner that is dependent on viral replication. Furthermore, the high significance of protein upregulation in the absence of significant mRNA induction suggests a component of translational regulation.

### 3.3. Multiple GLUT Proteins Mediate Glucose Transport into Human CD4+ T Cells

**Figure 2:**
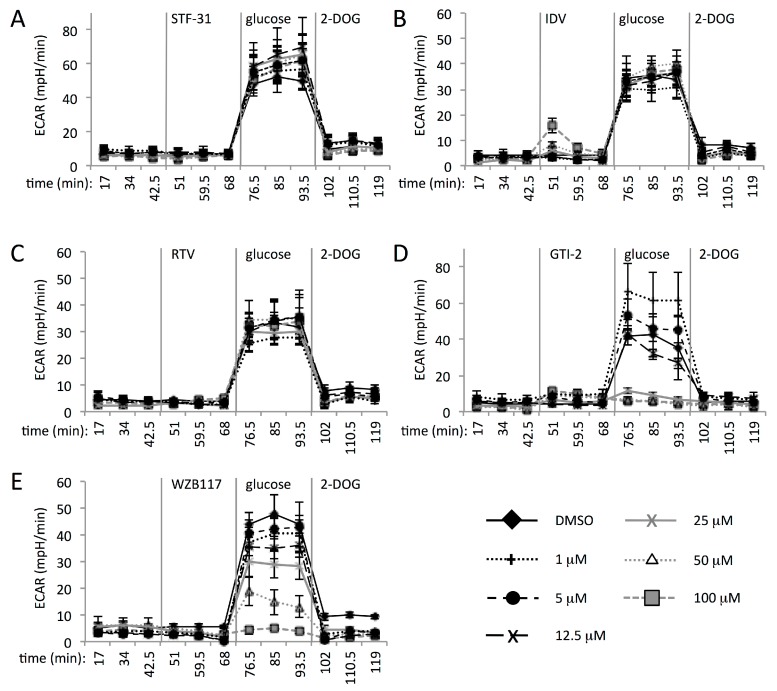
Blocking glucose uptake by CD4+ T cells requires inhibitors that target multiple GLUT proteins. The extracellular acidification rate (ECAR in mpH per min) was determined in real time for activated CD4+ T cells in the absence of glucose, followed by sequential injection of GLUT inhibitor at 0, 1, 5, 12.5, 25, 50, and 100 μM; glucose (1 g/L); and 2-deoxyglucose (4.5 g/L). (**a**) STF-31; (**b**) Indinavir (IDV); (**c**) Ritonavir (RTV); (**d**) GTI-2; (**e**) WZB117. The data shown are representative experiments from replicates with three to five different donors.

As we detected the expression of multiple GLUT proteins in activated human CD4+ T cells, we next sought to determine whether these contribute to glucose uptake (Figure 2). We assessed the production of lactic acid in response to provision of glucose in the presence of titrated amounts of glucose transporter inhibitors using the XF24 extracellular flux analyzer. The evaluation of GLUT inhibitors with embryonic kidney and lung carcinoma cell lines using the XF24 has been reported previously [50]. A limited selection of GLUT inhibitors are available, that were either designed/selected for this purpose or derive from antiretroviral drugs that have GLUT inhibitory activity as an unforeseen side effect. The drugs STF-31 [51], glucose transport inhibitor II (GTI-2) [52], and WZB117 [53,54,55] were developed as GLUT1 inhibitors, and the antiretroviral drugs indinavir and ritonavir have GLUT4 and GLUT1/GLUT4 inhibitory activity [56,57,58], respectively. It has previously been established that GTI-2 also inhibits GLUT3, GLUT4, and GLUT9, in addition to GLUT1 [52], which represents all of the GLUT proteins this drug has, to date, been tested against. WZB117 has been shown to inhibit GLUT3 equally effectively as GLUT1, and is in fact a more potent inhibitor of GLUT4 [59]. Thus, it seems plausible that both GTI-2 and WZB117 may inhibit additional glucose transporters.

After recording of the basal extracellular acidification rate (ECAR) in the absence of glucose, cells were pre-incubated with titrated amounts of the GLUT inhibitors for 24 min with subsequent injection of glucose into the culture at a final concentration of 1 g/L (Figure 2). This was followed by injection of an excess of 4.5 g/L 2-deoxyglucose (2-DOG) to fully inhibit glycolysis. Importantly, the glucose-dependent increase of the ECAR in the absence of drugs, as well as its inhibition by 2-DOG, confirmed that the acidification rate is attributable to the production and secretion of glucose-derived lactic acid. Intriguingly, we observed that the addition of STF-31 (GLUT1 inhibitor), indinavir (GLUT4 inhibitor), or ritonavir (GLUT1 and GLUT4 inhibitor), up to concentrations of 100 μM, failed to inhibit the ECAR of CD4+ T cells (Figure 2A–C). These concentrations exceed the reported IC50 of these compounds for their respective GLUT targets, being 1 μM for GLUT1 by STF-31 (MSDS), ≤21 μM for GLUT4 by indinavir, and 7–8 μM for GLUT1 and GLUT4 by ritonavir [56,60]. These results therefore demonstrate that inhibition of GLUT1 and GLUT4, either separately or in combination, does not fully abrogate glucose uptake into activated human CD4+ T cells. By contrast, the compounds GTI-2 and WZB117 inhibited the ECAR in a concentration-dependent manner, with complete inhibition being achieved at 25 μM for GTI-2, and 100 μM for WZB117. As indicated above, both GTI-2 and WZB117 are known to act on several GLUT proteins in addition to GLUT1, and share the ability to additionally inhibit GLUT3 and 4. Our comparison of inhibitors that target individual and multiple GLUT proteins therefore suggests that simultaneous inhibition of glucose transporters is required to block glucose uptake into activated human CD4+ T cells.

In addition to lactic acid production rates, the oxygen consumption rate (OCR) of the cells was recorded in parallel. In the case of STF-31, the OCR showed no response to addition of the drug, but a suppression of oxygen consumption after addition of glucose was evident in cultures that received the drug as well as in the control cultures (Figure S5A). This phenomenon is known as the Crabtree effect [61]; the reduction in oxygen consumption as the utilization of glucose for ATP production reduces the need for oxidative phosphorylation. The addition of ritonavir and indinavir likewise had no influence on cellular respiration [44]. By contrast, both GTI-2 and WZB117 affected CD4+ T cell respiration at all concentrations (Figure S5B,C). At 1 μM, GTI-2 had a stimulatory effect on the respiration rate, and a transient increase in respiration was also observed at 5 μM. At concentrations of 12.5 μM and above, GTI-2 inhibited cellular respiration. Inhibition of respiration was more pronounced with WZB117, which caused a reduction in the OCR at all concentrations tested. Part of the inhibition of the OCR by GTI-2 and WZB117 might be explained by the inhibition of glucose transport, as less glucose-derived pyruvate would be available to enter the citric acid cycle. However, the media used during the experiment (DMEM) contains glutamine and other amino acids that are available to support respiration in the absence of pyruvate. We therefore deem it more likely that both GTI-2 and WZB117 directly inhibit components of cellular respiratory metabolism in addition to their GLUT inhibitory activity.

### 3.4. Increased Glucose Consumption of HIV-1 Infected CD4+ T Cells

**Figure 3:**
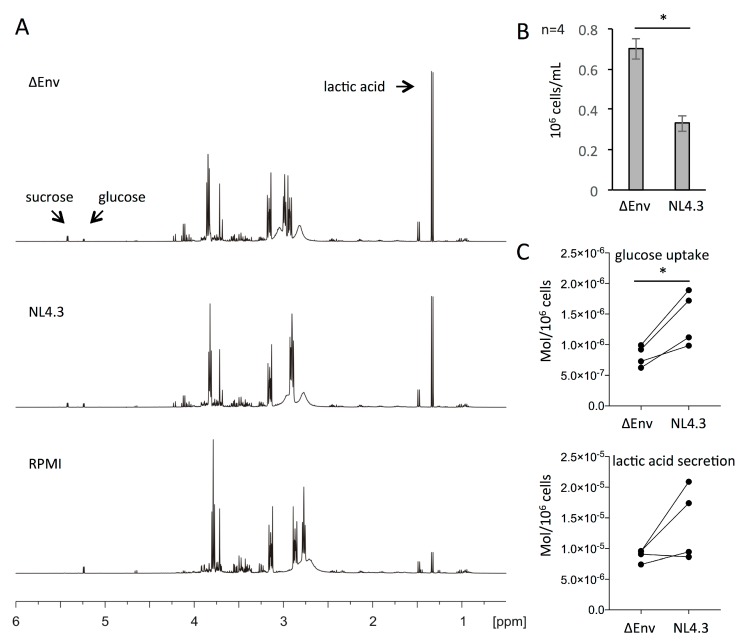
^1^H-NMR analysis of glucose consumption and lactic acid production of HIV-1 infected and uninfected CD4+ T cells. (**a**) Representative ^1^H-NMR spectra of the supernatant of CD4+ T cell cultures infected with HIV-1 NL4.3 or a non-infectious control (ΔEnv), as well as RPMI media without cells. Arrows indicate peaks corresponding to sucrose, glucose, and lactic acid; (**b**) The numbers of CD4+ T cells present in the cultures (ΔEnv or wt NL4.3) used for NMR analysis after 24 h; (**c**) Cell number-corrected glucose uptake and lactic acid production after 24 h of culturing CD4+ T cells infected with HIV-1 NL4.3 or the non-infectious ΔEnv control. Significance is indicated by * *p* ≤ 0.05.

We previously reported increased glycolytic activity in HIV-1 infected versus uninfected primary human CD4+ T cells by assessing the glucose-dependent acidification of culture media due to the secretion of lactic acid [15]. We sought to verify those observations by monitoring the consumption of glucose from the culture media of CD4+ T cells, infected or not with HIV-1, using ^1^H-NMR spectroscopy (Figure 3). We infected primary human CD4+ T cells with wt HIV-1 NL4.3 or the non-infectious ΔEnv NL4.3 preparations for 24 h, and equal amounts of infected and uninfected cells were seeded into RPMI media with 4.5 g/L of glucose. After the 24 h, the cell count in each culture was determined, and the culture media were harvested for NMR analysis (Figure 3A). Cultures of uninfected cells consistently yielded a higher cell count than those infected with HIV-1 (Figure 3B). This can be attributed to ongoing cell proliferation in the uninfected cultures, whereas the cells infected with HIV-1 are subject to virus-mediated cell cycle arrest and cell death [62]. Upon collecting the ^1^H-NMR spectra of the culture supernatants, peaks corresponding to glucose and lactic acid were readily identifiable by the use of reference spectra. We quantified the amount of glucose and lactic acid by their peak integrals, and calculated their respective consumption and production rates, which were then normalized to the number of cells present in the culture at the end of the experiment (Figure 3). This revealed a highly significant increase in glucose consumption in HIV-1 infected compared with uninfected cells (Figure 3C). The increased lactic acid production of HIV-1 infected cells failed to reach significance in this experiment (Figure 3C). This may be explained by the fact that not all of the glucose is fully metabolized to lactic acid. However, these results are in good agreement with our previous studies that demonstrated significant increases in the lactic acid production of HIV-1 infected cells using the XF24 extracellular flux analyzer [15]. Measurements of acidification using the XF24 instrument are performed over much shorter periods of time (min instead of days), during which it is easier to control cell numbers, and thereby the accuracy of the data.

Finally, we also identified a signal in the ^1^H-NMR spectra corresponding to sucrose, which was present in cultures that had received HIV-1 NL4.3 wt or ΔEnv inocula, but not in control media without cells or virus. The sucrose was introduced into the cultures by the virus preparations we used for infecting the cells, which had been purified over a sucrose cushion by ultracentrifugation. Importantly, the peaks corresponding to sucrose showed no evidence of consumption over time nor any difference between infected and uninfected cells. In summary, we demonstrated a greater rate of glucose consumption in HIV-1 infected compared with uninfected CD4+ T cells, which correlates with the elevated expression of GLUT1, 3, 4, and 6 proteins in the former. Attempts to further dissect the contributions of each individual GLUT protein to the HIV-mediated increase in glucose uptake were thwarted the off-target effects, as well as toxicity associated with the GLUT inhibitors in long term tissue culture. In addition, targeting of individual GLUT proteins with either Accell siRNA or shRNA-expressing retroviral vectors did not achieve sufficient knockdown to perform meaningful analyses. Of note, several studies have reported only marginal knockdown of GLUT proteins by RNAi strategies [63,64,65,66].

### 3.5. HIV-1 Infected Cells Have Increased Hexokinase Activity That Is Mediated by Upregulation of HK1

Having established that HIV-1 infected CD4+ T cells have greater glucose uptake and consumption rates than uninfected cells, we aimed to identify specific components of the glycolysis pathway that are differentially active in infected and uninfected cells. We therefore collected lysates from primary human CD4+ T cells that were treated with HIV-1 NL4.3 wt or the ΔEnv control for the analysis of enzyme kinetics, by spectroscopic determination of NAD(P)H (nicotinamide adenine dincleotide/phosphate) production or consumption rates as coupled reactions (see Section 2). As in our preceding experiments, infected samples were included in the analysis if a minimum of 60% of the cells expressed p24^Gag^ as determined by flow cytometry.

**Figure 4:**
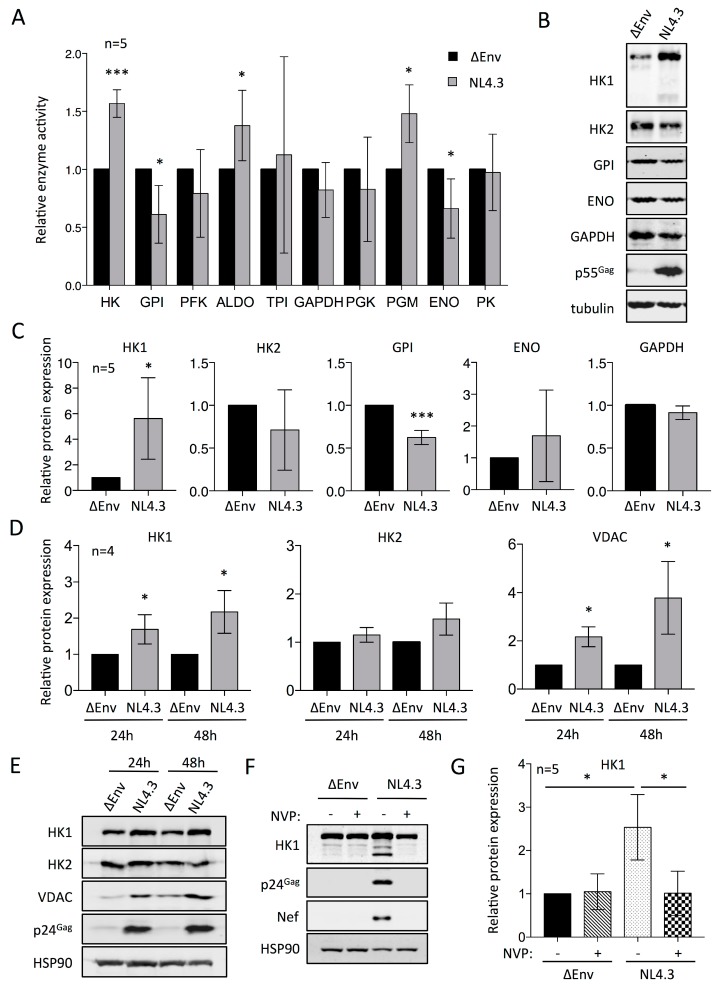
Relative enzymatic activity and expression levels of glycolytic factors in uninfected and HIV-1 infected CD4+ T cells. (**a**) Relative activity of glycolytic enzymes in lysates from uninfected (∆Env) and HIV-1 infected (NL4.3) cells after 48 h of infection: hexokinase (HK), glucose-6-phosphate isomerase (GPI), phosphofructokinase (PFK), aldolase (ALDO), triose phosphate isomerase (TPI), glyceraldehyde-3-phosphate dehydrogenase (GAPDH), phosphoglycerate kinase (PGK), phosphoglycerate mutase (PGM), enolase (ENO), and pyruvate kinase (PK); (**b**) Representative Western blotting analysis of expression of glycolytic enzymes in cell lysates from (**a**); (**c**) Relative abundances of glycolytic enzymes from the Western blotting analysis shown in (**b**) from five donors. Protein abundances were quantified relative to tubulin expression and values normalized to the amounts present in cells treated with ΔEnv NL4.3; (**d**) Time course analysis of changes in protein expression of HK1, HK2, and VDAC at 24 and 48 h following infection with HIV-1 wt or ΔEnv NL4.3. Protein abundances were quantified from Western blots relative to HSP90 expression and values normalized to the amounts present in cells treated with ΔEnv NL4.3; (**e**) Representative Western blotting analysis of protein expression for the data shown in (**d**); (**f**) Representative Western blotting analysis of the expression of HK1, p24^Gag^, Nef, and HSP90 in CD4+ T cells infected with HIV-1 wt or ΔEnv NL4.3 in the presence and absence of nevirapine (NVP) at 48 h after infection; (**g**) Quantified data of HK1 expression relative to HSP90 and normalized to the amounts present in cells treated with ΔEnv NL4.3 from the Western blotting analysis shown in (**f**), for five donors. Significance is indicated by * *p* ≤ 0.05 and *** *p* ≤ 0.001.

The analysis of enzyme kinetics revealed both increased and decreased activities of some glycolytic enzymes in the lysates of HIV-1 infected compared with uninfected cells (Figure 4A). In particular, the activities of hexokinase (HK), aldolase (ALDO), and phosphoglycerate mutase (PGM) were increased, while the activities of glucose-6-phosphate isomerase (GPI) and enolase (ENO) were decreased in HIV-1 infected cells in a statistically significant manner. Of these, the single most significant and greatest change in activity was observed with hexokinase, whose activity in HIV-1 infected cells was 1.5-fold higher than in uninfected cells.

We next set out to assess the expression levels of these glycolytic enzymes in the same samples used for the kinetic analysis of enzyme activity. A complicating factor in these studies is that many enzymes in glycolysis have multiple isozymes, some of which are known to have tissue-specific expression, whilst for others, this has not yet been comprehensively determined. We therefore focused our analyses on HK (isozymes 1–4), ENO (alpha subunit), GPI and GAPDH. The latter two enzymes have only a single known isoform. We already observed in our preceding experiments that only isoforms 1 and 2 of HK are expressed in activated human CD4+ T cells (Figure S4A), and isoforms HK3 and HK4 likewise remained undetectable in HIV-1 infected CD4+ T cells. We observed that low levels of HK1 were present in resting cells with subsequent approximate 3-fold upregulation at day 1 after activation, which reached levels of up to 5-fold upregulation by day 3 after activation (Figure S4A). HK2 showed a greater upregulation in response to activation of approximately 10-fold at day 1, and up to 30-fold by day 3 after activation (Figure S4A). Interestingly, we observed a consistent upregulation in the expression of HK1, but not HK2, in the HIV-1 infected cells compared with uninfected cells (Figure 4B,C). In accordance with its decreased activity in HIV-1 infected cells, the expression of GPI was reduced compared with uninfected cells (Figure 4B,C). The expression of ENO was quite variable, and did not correlate with its decreased activity in HIV-1 infected cells (Figure 4A–C). GAPDH expression levels were similar in infected and uninfected cells, which is in accordance with its unaltered enzymatic activity after infection with HIV-1. We also assessed the expression of G6PDH in HIV-1 infected versus uninfected cells (Figure S5D). G6PDH acts on glucose-6-phosphate, the product of hexokinase, and targets it to the pentose phosphate pathway (PPP) as 6-phospho-d-glucono-1,5-lactone. The expression of G6PDH was consistently and significantly downregulated in HIV-1 infected compared with uninfected CD4+ T cells.

The elevated hexokinase activity and HK1 expression levels were the most significant alterations to glycolysis in response to infection of CD4+ T cells with HIV-1 that we observed in the course of our investigations. As the enzyme kinetics and expression were initially assessed in cells that had been infected with HIV-1 for 48 h, we sought to determine if these changes might be detectable earlier after infection. We therefore compared the expression levels of HK1, HK2, and VDAC in HIV-1 infected and uninfected CD4+ T cells at 24 and 48 h after infection (Figure 4D,E). VDAC is a voltage dependent anion channel that is located in mitochondrial membranes, and conditionally participates in a physical interaction with HK1/2 that enhances their activity and additionally regulates the balance between pro- and anti-apoptotic signaling [67]. Western blotting analysis revealed that the upregulation of HK1 in HIV-1 infected cells is already apparent at 24 h after infection, and this coincides with upregulation of VDAC (Figure 4D,E). By contrast, HK2 was not significantly upregulated at 24 or 48 h after infection, as is consistent with our previous results (Figure 4B,C). Comparison of the expression of HK1 in HIV-1 infected cells treated or not with nevirapine revealed that its upregulation is dependent on virus replication. (Figure 4F,G).

**Figure 5:**
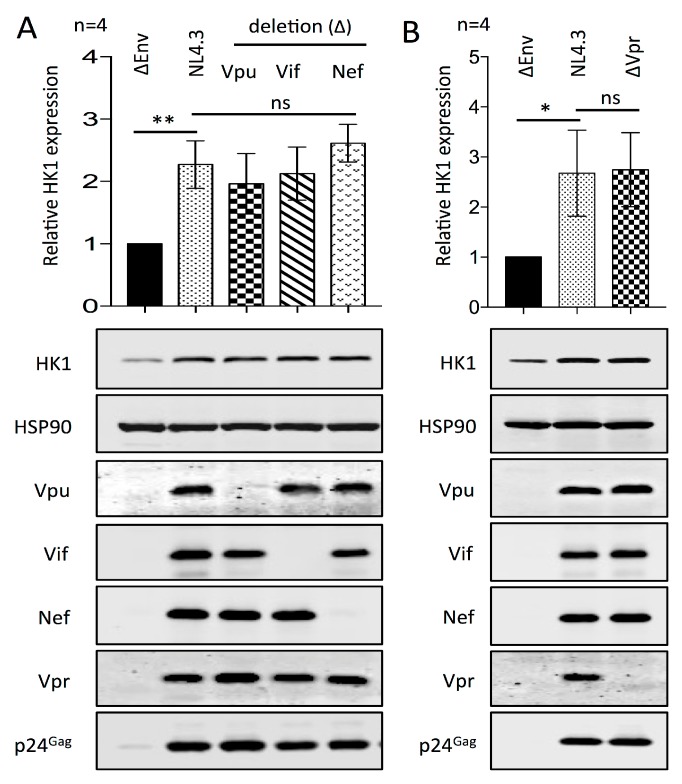
HIV-1 accessory proteins are not required for the upregulation of HK1 in CD4+ T cells infected with HIV-1 NL4.3. (**a**) Western blotting analysis of CD4+ T cells infected with HIV-1 wt NL4.3 or mutant viruses that have the Env, Vpu, Vif, or Nef genes deleted. Quantified data, that are shown above in a representative Western blot, are relative to HSP90 expression and normalized to the amounts present in cells treated with ΔEnv NL4.3, for four donors; (**b**) Western blotting analysis of CD4+ T cells infected with HIV-1 wt NL4.3 or mutant viruses that have the Env or Vpr genes deleted. Quantified data, that are shown above in a representative Western blot, are relative to HSP90 expression and normalized to the amounts present in cells treated with ΔEnv NL4.3, for four donors. Significance is indicated by * *p* ≤ 0.05 and ** *p* ≤ 0.01.

HIV-1 encodes four accessory proteins, Vif, Vpr, Vpu, and Nef, that have the capacity to modulate the expression of host-encoded proteins through mechanisms ranging from transcriptional control to ubiquitin-mediated proteolysis [68,69]. In order to assess whether any of the HIV-1 accessory proteins mediate the upregulation of HK-1, we performed Western blotting analysis of cells infected with wt NL4.3 HIV-1, as well as NL4.3 molecular clones from which the Vif, Vpr, Vpu, or Nef genes were deleted (Figure 5). Upregulation of HK1 was apparent in the infected cultures compared with the ΔEnv NL4.3 non-infectious control, regardless of the deletion of any of the accessory proteins. We therefore conclude that the HIV-1 accessory proteins are not required to mediate the upregulation of HK1, and by extension, glycolysis, upon infection of CD4+ T cells.

## 4. Discussion

We have investigated the expression and activity of glycolytic factors in human CD4+ T cells upon activation and in response to infection with HIV-1. CD4+ T cell activation is well documented to coincide with an upregulation of glycolytic metabolism that is widely attributed to increased GLUT1-mediated glucose transport [7,8,10,11,16,26,35]. Although some previous studies indicated that other GLUT proteins might be expressed in human and murine lymphocytes [31,32,33,34,35], the identity of GLUT proteins that are involved in human CD4+ T cell activation and differentiation has not been previously addressed. We showed that in addition to GLUT1, there was considerable upregulation of GLUT3, GLUT4, and GLUT6 in response to activation at the mRNA and protein level. Persistence of activation-dependent GLUT expression in the presence of the transcription inhibitor, actinomycin D, demonstrated that these proteins are regulated at the translational as well as the transcriptional level. We also observed that GLUT3 was moderately enriched in the human Th17 subset, which is in line with GLUT3 enrichment in murine Th17 cells [29]. That the activation of human CD4+ T cells leads to the expression of multiple glucose transporters in addition to GLUT1 appears consistent with the absence of any reported immune-pathology in individuals suffering from GLUT1-deficiency, which is primarily associated with neuronal dysfunction [70]. We furthermore showed that the activation of human CD4+ T cells coincides with the upregulation of the hexokinases HK1 and HK2, but not HK3 and HK4, the latter being undetectable in resting as well as activated CD4+ T cells.

Importantly, we demonstrated that the expression of multiple GLUT proteins is likely to contribute to glucose uptake in activated human CD4+ T cells, as inhibitors that target GLUT1 (STF-31), GLUT4 (indinavir), or both GLUT1 and GLUT4 (ritonavir) failed to affect glucose-dependent lactic acid production. On the contrary, the inhibitors WZB117 and GTI-2 effectively inhibited glycolysis in activated CD4+ T cells, and these compounds are known to inhibit several GLUT proteins in addition to GLUT1. Our experiments furthermore revealed that both these drugs have previously unreported inhibitory activity with regards to cellular oxygen consumption. Indeed, we observed that both WZB117 and GTI-2 possess strong anti-proliferative properties, as well as considerable cell toxicity in resting as well as activated cells in long term cell culture [44].

In accordance with our previous studies that reported increased glucose-dependent lactic acid production of HIV-1 infected CD4+ T cells compared with uninfected cells [15], we formally demonstrated that HIV-1 infected cells also have increased glucose uptake by ^1^H-NMR studies. This increased rate of glucose uptake in HIV-1 infected cells was mirrored by the upregulation of the glucose transporters GLUT1, 3, 4, and 6 in a replication-dependent manner. We note that upregulation of GLUT1 and GLUT3 mRNA, as well as protein, was previously reported on infection of the lymphocytic cell line H9 with HIV-1, but GLUT4 was reportedly undetectable in that cell line, and the presence of GLUT6 was not investigated [71]. We previously reported that the metabolic phenotype of T cell lines after infection with HIV-1 does not reproduce the elevation of glycolysis that is evident in HIV-1 infected primary CD4+ T cells [15]. Evidently, there are also differences in the identity of glucose transporters that immortalized lymphocytic cell lines express, and which may contribute to this discrepancy.

Prompted by the upregulation of glucose transporters and increased glucose uptake, we also compared the activity of all enzymes in the glycolysis pathway in HIV-1 infected and uninfected cells. This revealed a highly significant increase in hexokinase activity that correlated with increased expression of HK1, but not HK2, in HIV-1 infected cells. Thus, unlike the concerted overexpression of all glucose transporters present in CD4+ T cells that we observed in response to infection with HIV-1, there is specificity in the upregulation of HK1. The upregulation of HK1 coincided with upregulation of VDAC that is known to engage in a functional interaction with hexokinases, which enhances their enzymatic activity [72,73]. Upregulation of VDAC was previously observed in a proteomic analysis of HIV-1 infected cells [74]. Importantly, the upregulation of HK1 was dependent on active viral replication, but independent of the expression of the HIV-1 accessory genes Vpu, Vpr, Vif, and Nef. Intriguingly, these data contrast with a report that demonstrated increased HK1 expression in HIV-1 infected PBMCs that coincided with decreased hexokinase activity and the upregulation of HK1 in U936-derived macrophages that were stably transduced with HIV-1 Vpr [75]. The reason for these discrepancies are currently unclear, but may relate to cell-type specific metabolic manipulation of T cells and macrophages by HIV-1 [41,42].

Upregulation of glucose transporters and hexokinases has been reported in the case of other viral infections. For instance, infection with HCMV lead to the expression of GLUT4 in human fibroblasts that normally express GLUT1 when uninfected, and this resulted in increased glucose uptake [76]. The infection of human foreskin fibroblasts with DENV-2 resulted in increased glucose consumption and overexpression of GLUT1 as well as HK2 [77]. The increase in glycolysis of KSHV infected dermal microvascular endothelial cells coincided with the overexpression of GLUT1 and HK2 [78]. Infection of a variety of cell lines with EBV resulted in increased expression of GLUT1, GLUT4, and HK2 that also increased glycolysis [79]. All of these studies invoked models of viral dependency on the elevated state of glycolysis, and provided evidence in support thereof through inhibition of viral replication by, or the increased sensitization of infected cells to, metabolic inhibitors. Likewise, we have previously demonstrated reduced virus production and pathogenicity upon the inhibition of glycolysis with galactose and 2-DOG [15].

However, the question remains whether the increase in glycolysis may represent a cellular response to viral infection in addition to, or instead of, a metabolic program conducive to virus replication. To date, we have been unable to unequivocally make that distinction. Indeed, a recent study has reported that HK acts as an innate immune sensor in macrophages and dendritic cells by recognizing cytoplasmic *N*-acetylglucosamine (GlcNac) released after the degradation of bacterial peptidoglycans, thereby activating the NLRP3 inflammasome [80]. However, the recognition of GlcNac was associated with inhibition of HK activity, which is opposite to what we have observed with regards to HK activity in HIV-1 infected CD4+ T cells. Although GlcNac is also an important substrate for eukaryotic protein glycosylation, including HIV-1 Env [81,82], in mammalian cells, it is thought to be primarily restricted to the ER/Golgi, and covalently linked to UDP nucleotide carriers.

In this context, it is of interest to note that the boundary between beneficial and inhibitory host factors of virus replication may generally be more blurred than is widely appreciated. For example, the cellular restriction factor APOBEC3G can limit HIV-1 infection by inducing G-to-A hypermutations in nascent viral DNA, and this is counteracted by Vif-mediated and ubiquitin-dependent degradation of APOBEC3G [83]. However, it has been shown that naturally occurring Vif alleles with suboptimal capacity to target APOBEC3G provide a window of opportunity to greater sequence diversity in the viral quasispecies, including mutations resulting in ART-resistance, even in the absence of drug pressure [84]. Similarly, tetherin restricts the release of newly budded virions by physical attachment to the plasma membrane of producer cells, and this is counteracted by the Vpu protein of HIV-1 through ubiquitin-dependent degradation of tetherin [85]. However, it has also been reported that tetherin can promote cell-to-cell transfer between T cells by facilitating the assembly of virological synapses [86]. It may well be the case that the metabolic interplay between HIV-1 and CD4+ T cells we describe in this work similarly represents a balance between processes that are beneficial to virus replication and those that serve to protect the host cells from lytic infection.

## 5. Conclusions

We have, for the first time, investigated the full range of glucose transporters that are expressed in human CD4+ T cells on activation and infection with HIV-1, being GLUT1, 3, 4, and 6. We demonstrated that HIV-1 infected CD4+ T cells have increased glucose uptake and concerted overexpression of this full set of glucose transporters compared with uninfected cells. The increase in glucose uptake of HIV-1 infected CD4+ T cells was furthermore shown to coincide with considerable metabolic modulation, as demonstrated by determining the enzymatic activities of all proteins in the glycolysis pathway. This revealed a marked increase in hexokinase activity in infected compared with uninfected cells that correlated with increased expression of HK1, but not HK2. The HIV-1-dependent elevation of HK1 expression was replication dependent, but independent of the expression of the viral accessory proteins Vif, Vpu, Vpr, and Nef. Whilst it therefore remains to be determined which viral factors mediate the elevated glucose consumption in HIV-1 infected cells, it is clear that early steps in glucose metabolism are affected, as both glucose import and phosphorylation are thought to be regulatory steps that commit glucose to cellular metabolism. This modulation of glucose metabolism in HIV-1 infected CD4+ cells, and their increased sensitivity to metabolic inhibition, are consistent with viral dependency on metabolic resources of the host, though a cellular response to infection cannot be ruled out. With the increased interest in targeting metabolic pathways for therapeutic strategies [8,87], our work suggests that it may be feasible to target factors of glucose metabolism subject to modulation by HIV-1, in order to shift the balance between virus replication and host responses in favor of host survival.

## Supplementary Materials

The following are available online at http://www.mdpi.com/1999-4915/10/3/114/s1, Figure S1. The expression of GLUT proteins in resting versus activated human CD4+ T cells, Figure S2. Expression of GLUT proteins in CD45RA+ and CD45RO+ CD4+ T cells upon activation, Figure S3. Activated human CD4+ Th17 cells show enriched expression of GLUT3, Figure S4. The upregulation of glucose transporters in response to activation of human CD4+ T cells and infection with HIV-1. Figure S5. GLUT inhibitors GTI-2 and WZB117 have additional inhibitory effects on cellular oxygen consumption.
