# Supplementary figures and images for: Upregulation of Glucose Uptake and Hexokinase Activity of Primary Human CD4+ T Cells in Response to Infection with HIV-1
Source: Viruses. 2018 Mar 7;10(3):114. doi: 10.3390/v10030114 (PMC5869507; doi:10.3390/v10030114)

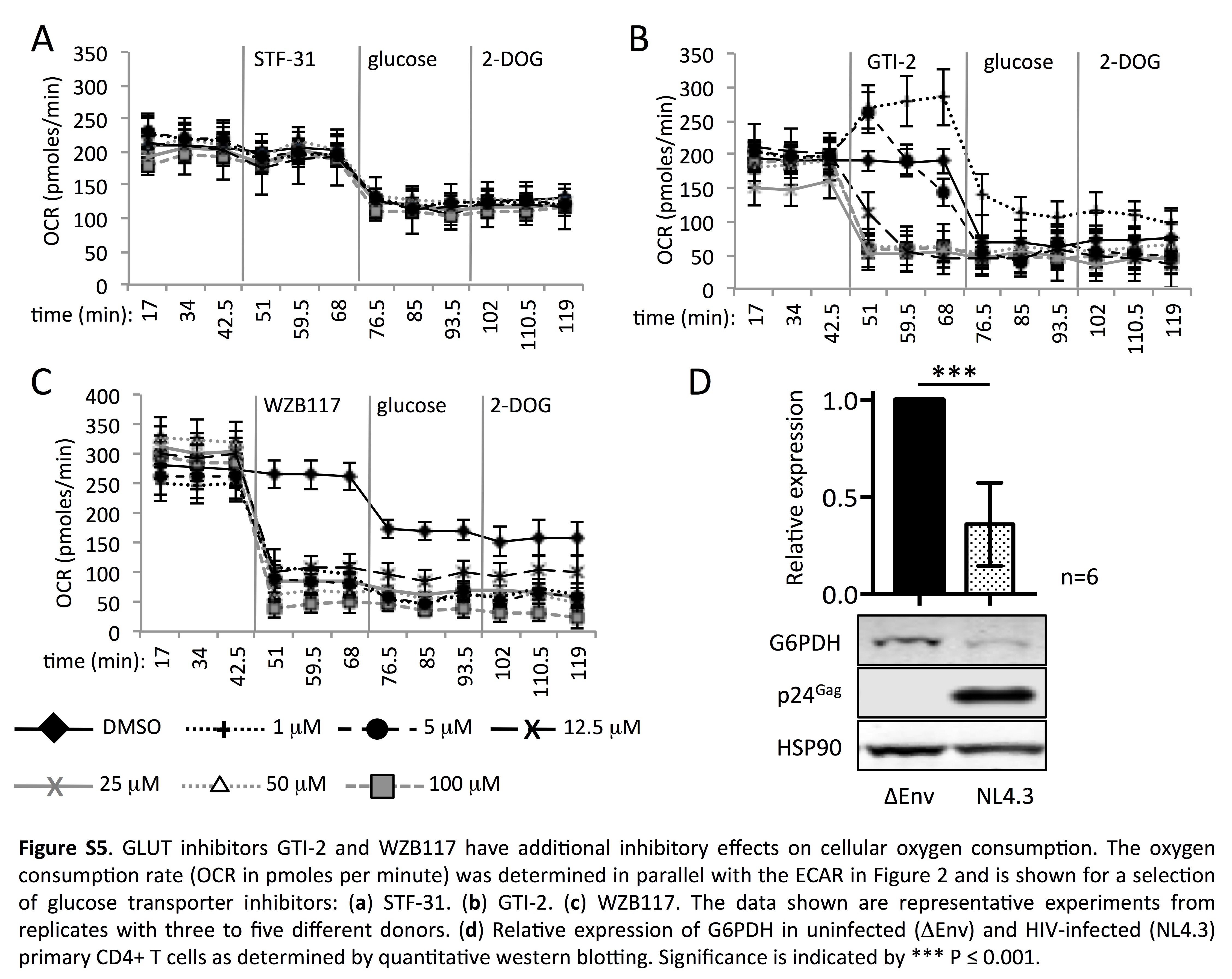

Supplement: Supplementary file 1 [file viruses-10-00114-s001.zip › mdip revised sup figures/MKW-S5.tiff]
